# Autoantibodies in Senear-Usher Syndrome: Cross-Reactivity or Multiple Autoimmunity?

**DOI:** 10.1155/2012/296214

**Published:** 2012-12-20

**Authors:** María Elena Pérez-Pérez, Esperanza Avalos-Díaz, Rafael Herrera-Esparza

**Affiliations:** Department of Immunology, Universidad Autónoma de Zacatecas, Chepinque 306, Colonia Lomas de la Soledad, 98040 Zacatecas, ZAC, Mexico

## Abstract

Senear-Usher syndrome or pemphigus erythematosus is a pathology that overlaps clinically and serologically with pemphigus foliaceus and lupus erythematosus. Skin biopsies of patients with pemphigus erythematosus reveal acantholysis and deposits of immunoglobulins in desmosomes, and they are positive in the lupus band test. In the present paper, we determined whether the autoantibodies associated with pemphigus erythematosus targeted a single antigen or multiple antigens as a result of the stimulation of independent B cell clones. Our present paper demonstrates that patients with pemphigus erythematosus produce both antiepithelial antibodies specific for desmoglein 1 and 3 and antinuclear antibodies specific for Ro, La, Sm, and double-stranded DNA antigens. After eluting specific anti-epithelial or anti-nuclear antibodies, which were recovered and tested using double-fluorescence assays, a lack of cross-reactivity was demonstrated between desmosomes and nuclear and cytoplasmic lupus antigens. This result suggests that autoantibodies in pemphigus erythematosus are directed against different antigens and that these autoantibodies are produced by independent clones. Given these clinical and serological data, we suggest that pemphigus erythematosus behaves as a multiple autoimmune disease.

## 1. Background

Pemphigus is a rare organ-specific autoimmune disease characterised by blistering of the skin and mucous membranes. Pemphigus is histopathologically characterised by acantholysis [1] and encompasses a spectrum of conditions, including the following: pemphigus vulgaris, pemphigus foliaceus, pemphigus vegetans, paraneoplastic pemphigus, and pemphigus erythematosus (PE), which is also known as Senear-Usher syndrome. In PE, the blistering coincides with a seborrheic erythematous rash resembling the rash associated with lupus. Serologically, PE patients have autoantibodies, which is similar to individuals with pemphigus foliaceus and cutaneous lupus erythematosus. Likewise, the skin immunopathology of pemphigus erythematosus is characterised by acantholysis with immunoglobulin deposition in desmosomes and at the dermal-epidermal junction (lupus band test). Histology and serological marker antidesmoglein 1 of pemphigus foliaceus and PE are the same [2].

The first patient with this disease was reported by Ormsby and Mitchell, and, soon after, Senear and Usher described a group of 11 patients with pemphigus who shared clinical features of lupus erythematosus and whose biopsies revealed acantholysis [3]. Subsequently, pemphigus erythematosus was proposed to be a clinical variant of pemphigus foliaceus. Currently, most clinicians consider the disease to be a benign type of pemphigus foliaceus that includes symptoms and serological characteristics of lupus erythematosus. 

The clinical hallmarks of PE are seborrheic lesions in the nose, nasolabial folds, and malar areas resembling the “butterfly” distribution of lupus. Lesions may also affect the preauricular region. Hyperkeratotic scars with erythema and superficial blisters can be present on the chest [4]. The oral mucosa, pharynx, and vulva are not involved.

Autoantibodies are directly involved in the pathogenesis of pemphigus, whereas in lupus, the autoantibodies are associated with glomerulonephritis. The presence of these autoantibodies may precede the clinical presentation, and a high autoantibody titer is usually associated with inflammatory flare-ups. The pathogenic role of antidesmoglein 1 antibodies in pemphigus foliaceus has been demonstrated by the blister production in experimental animals after injection of pemphigus foliaceus IgG [5, 6]; on the other hand, there are few reports that provide the frequency of antinuclear antibodies in pemphigus, however its frequency and pathogenic role in PE are still unknown [7, 8].

Despite this knowledge, antigenic triggering is not fully understood in either disease. There are some clues that suggest that environmental and other cell stress-related factors might trigger autoantibody production; however, the factors that cause multiple autoimmunity remain to be identified. Because PE patients have pemphigus and lupus autoantibodies and because the disease behaves clinically similar to benign pemphigus and cutaneous lupus, the major questions that we attempted to address were the following. Do pemphigus erythematosus autoantibodies target a single antigen? If so, such polyreactivity might result from cross-reactivity. Alternatively, do PE autoantibodies result from stimulation by different antigens? In this case, the polyreactivity could result from intermolecular epitope spreading.

## 2. Materials and Methods

### 2.1. Sera

Samples collected in our laboratory between 1995 and 2008 from patients with a clinical diagnosis of pemphigus were used in the present investigation. The initial inclusion criterion was the presence of both antiepithelial (AEA) and antinuclear antibodies (ANA), as determined by immunofluorescence. 

From an initial group of 52 positive patients, 10 were chosen because they had titers of at least 1 : 160 for both pemphigus and lupus autoantibodies. We chose this ANA titer cutoff because higher titers are internationally accepted as positive results. Additionally, 10 healthy controls matched by age and sex were included. This group was included because cow nose is not a common antigenic source for antinuclear antibody determination.

The ten sera selected for our investigation were obtained from patients with a diagnosis of pemphigus erythematosus. Eight patients were women and two males with a mean age of 45 years. Clinically, these patients had blisters and erythematous and seborrheic lesions distributed along the malar region, retroauricular areas, and the upper part of the chest. All of the patients had positive biopsies for pemphigus, with intraepidermal blisters and acantholytic cells. None of these patients had renal, cardiopulmonary, or CNS involvement.

### 2.2. Assessment of Antiepithelial Antibodies by Indirect Immunofluorescence

Fresh cow nose obtained from the slaughter house was used as a source to detect pemphigus antigens. The tissue was transported on ice to the laboratory, embedded in Tissue-Tek OCT, and frozen at −20°C. Tissue samples were cut at 4 *μ*m using a cryostat (−20°C), and the slices were fixed on slides [9]. Pemphigus sera were tested at dilutions of 1 : 80 to 1 : 2560. Serum samples were incubated with the antigenic substrate for 30 minutes in a moist chamber, and the slides were washed three times in PBS (pH 7.2). This step was followed by a 30-minute incubation with polyvalent FITC-labelled rabbit anti-human antiserum (Sigma-Aldrich, St Louis, MO, USA). After three additional PBS washes, the slides were mounted with glycerol-PBS (9 : 1) and evaluated using an Olympus B-Max BX-40 fluorescence microscope by two independent observers in a blind manner.

### 2.3. Antinuclear Antibodies

Commercial HEp-2 cells (Immuno Concepts NA, Ltd, Sacramento, CA) on slides were incubated for 30 minutes with serum diluted from 1 : 160 to 1 : 1280, followed by three PBS washes and a 30-minute incubation with FITC-labelled rabbit anti-human polyclonal gamma globulin (IgG, IgA, and IgM, Sigma). After another round of washing, the slides were mounted in glycerol-PBS and evaluated with fluorescence microscopy [10].

### 2.4. Digestion with Nucleases or Trypsin

The cross-reactivity of autoantibodies in pemphigus erythematosus sera was examined in two assays. (1) To eliminate the nuclear antigens present in the cow nose tissue, the tissues were digested by a 30-minute incubation with a nuclease mixture (0.25% DNAse/RNAse I, Sigma, St Louis, MO, USA) at 37°C. After digestion, the slides were washed in PBS three times, followed by a 30-minute incubation with a 1 : 160 dilution of patient serum. After washing, the slides were further incubated with a FITC-labelled goat anti-human IgG. Finally, the levels of nuclear staining and fluorescence in the intercellular spaces were evaluated. (2) To eliminate the desmosomes, the slides were digested for 1 minute with 0.5% trypsin, and the desmosome antigens were removed by a brief wash with SSC buffer (NaCl 150 mM and NaCit 15 mM). Then, the level of antinuclear reactivity was evaluated. Two positive controls were included in this experiment to ensure the elimination of antigens by the enzymes. One positive control was serum from an individual with systemic lupus erythematosus with high ANA (1 : 5120) and anti-DNA titers (1 : 1280). This serum sample was tested in cow nose digested with nucleases. The other positive control was serum from an individual with pemphigus vulgaris who was AEA positive (1 : 2560). This serum sample was tested on cow nose digested with trypsin.

### 2.5. Affinity-Purified Antibodies

Each serum sample was incubated for 30 minutes with cow nose or HEp-2 cells that had been digested with trypsin or nucleases. Then, the slides were washed in PBS three times, and specifically bound antibodies were eluted with 0.2 M glycine-HCl, pH 2.8. The eluate was neutralised with 1 M Tris, pH 9.5 [11], and the eluted antibodies were concentrated with a Centricon centrifugal device with a 30-kDa molecular weight cut-off (Millipore, Billerica, MA, USA). Then, the affinity-purified antiepithelial and antinuclear antibodies were used to perform the double fluorescence labelling and ELISA assays described below. 

### 2.6. Double-Fluorescence Labelling Assays

To assess the specific reactivity of pemphigus erythematosus sera with desmosomal junctions, the affinity-purified antiepithelial antibodies were incubated for 30 minutes with undigested cow nose tissue. The samples were washed three times with PBS, and the bound antibodies were then tagged with a goat anti-human IgG FITC-labelled antibody during a 30-minute incubation. After this incubation, another round of PBS washing was performed. To assess the specific nuclear reactivity, the slides were incubated for 30 minutes with affinity-purified antinuclear antibodies, and after washing with PBS, a red nuclear tag was activated with a 30-minute incubation with Texas red-labelled goat anti-human IgG. Finally, the slides were mounted and evaluated under a fluorescence microscope using the appropriate filters for the dyes. In these experiments, the desmosomes were stained green and the nuclei were stained red.

### 2.7. Fine Antigenic Specificity

The affinity-purified antiepithelial antibodies in each serum sample were tested for reactivity against desmoglein 1 and 3 using commercial ELISA kits (MBL, International, Woburn, MA, USA). Additionally, the antinuclear reactivity to Ro, La, Sm/RNP and phospholipids was assessed by ELISA (Euroimmun AG, Lübeck, Germany). The anti-DNA activity was assessed by indirect immunofluorescence using the *Crithidia luciliae* assay. 

## 3. Results

### 3.1. Sera

**Figure 1:**
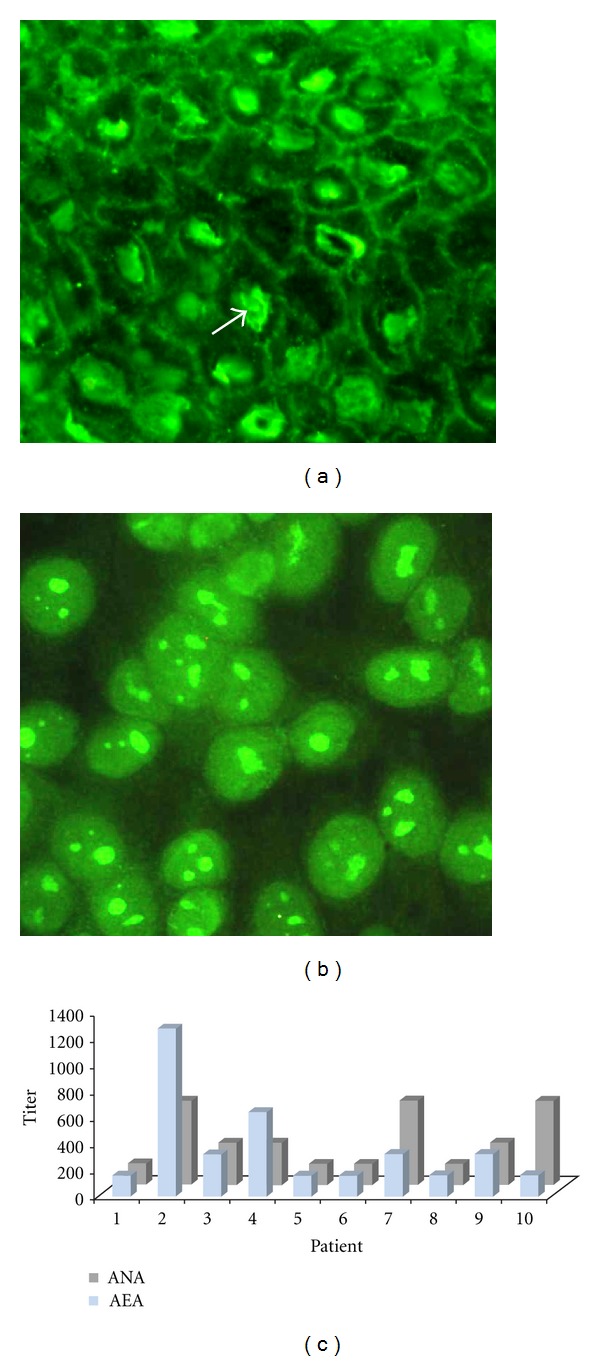
(a) Indirect immunofluorescence in cow nose tissue showing a honeycomb fluorescence pattern along intercellular junctions of keratinocytes that is characteristic of pemphigus and a nuclear fluorescence pattern for ANA. (b) The same serum sample was tested in HEp-2 cells, resulting in a homogeneous nuclear and nucleolar pattern. (c) Graph of individual titers of each serum sample.

All of the sera were positive for pemphigus antiepithelial antibodies at variable titers, and the antibodies in eight sera reacted with desmogleins (Dsg). With regard to lupus specificity, all of the sera tested positive for antinuclear antibodies at titers above 1 : 160 on HEp-2 cells. The dominant fluorescence pattern was fine speckles (in six sera). One serum sample had a homogeneous nucleolar pattern, and the remaining sera sample exhibited diffuse nuclear fluorescence (Figure 1).

### 3.2. Serum Reactivity after Antigenic Source Digestion with Enzymes

**Figure 2:**
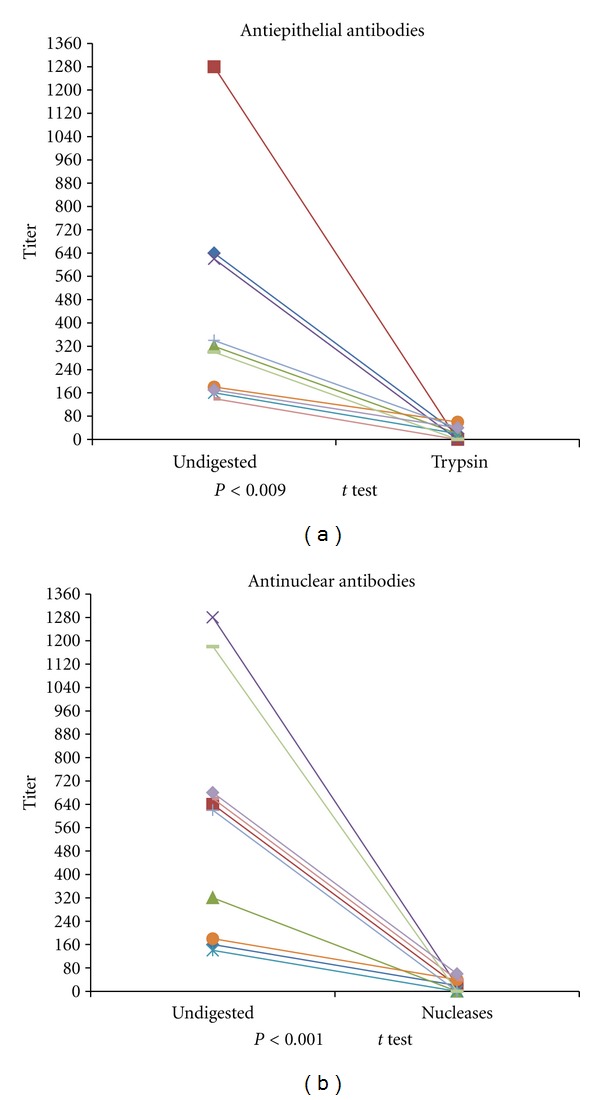
Antigenic digestion of cow nose using enzymes. (a), trypsin digestion abrogated the antiepithelial antibody (AEA) titers. (b), nuclease (RNAse/DNAse) digestion abrogated the antinuclear antibody (ANA) titer, as demonstrated by indirect immunofluorescence.

The antinuclear reactivity of pemphigus erythematosus patient sera was abrogated in HEp-2 cells digested with nucleases. The nuclear fluorescence disappeared in the cow nose cells, but fluorescence was still present in the intercellular spaces. In contrast, the intercellular reactivity disappeared but the nuclear fluorescence remained positive in cow nose treated with trypsin. Finally, in a set of cow nose tissue samples double digested with trypsin and nucleases all fluorescence was abrogated. Control experiments using high ANA-titer lupus serum were negative for cow nose digested with nucleases. In addition, the pemphigus vulgaris serum with a high AEA titer was negative with trypsin-digested cow nose. The results of this experiment demonstrate the ability of the digestion procedure to eliminate these antigens and that one serum sample can have two different specificities: one that is antiepithelial and another that is antinuclear (Figure 2). 

### 3.3. Double-Fluorescence Assays with Affinity-Purified Antibodies

**Figure 3:**
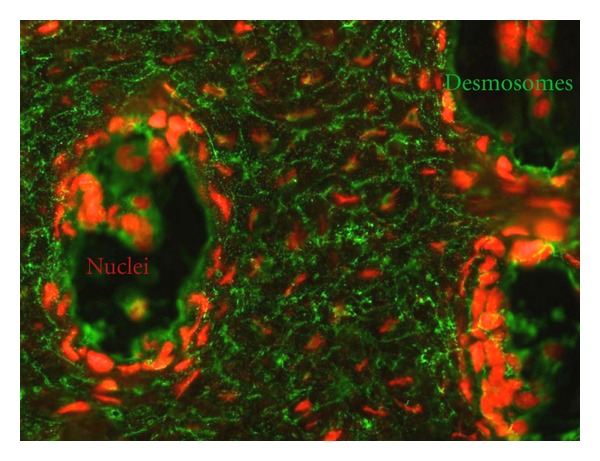
Double fluorescence assay using cow nose tissue. Cellular junctions were tagged in green (FITC) with labelled antiepithelial antibodies, and nuclei were tagged in red with labelled antinuclear antibodies (Texas red).

The affinity-purified antiepithelial antibodies tested on undigested cow nose and tagged in green were visualised in the intercellular spaces, producing a honeycomb fluorescence pattern characteristic of pemphigus. The affinity-purified antinuclear antibodies tagged in red were bound along the nuclei of keratinocytes. The merged image in Figure 3 shows fluorescence in two colours.

### 3.4. Fine Specificity of Affinity-Purified Antibodies

To determine the molecular specificity of the affinity-purified antibodies, they were tested with ELISA. Using this assay, we demonstrated that the antibodies eluted from the cow nose digested with nucleases exhibited anti-Dsg1 or anti-Dsg3 activity but not nuclear reactivity. Interestingly, eight sera were positive for both Dsg1 and Dsg3, but in two sera, despite AEA positivity, the fine specificity could not be determined. These autoantibodies were most likely directed against another desmosome component. 

**Figure 4:**
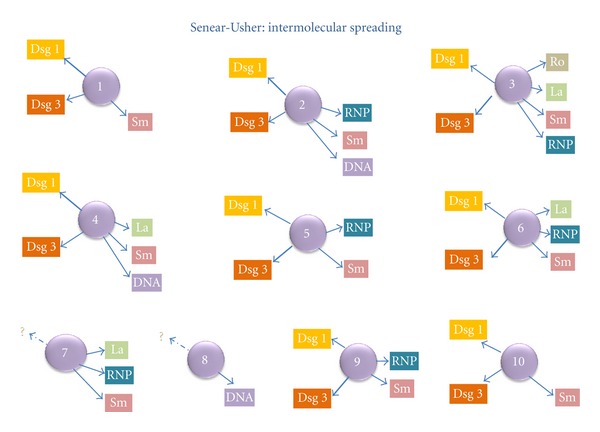
Multiple molecular reactivity of pemphigus erythematosus patient sera to desmosome and ribonucleoprotein components. This result may indicate intermolecular antigen spreading.

Similarly, the affinity-purified antibodies eluted from cow nose digested with trypsin displayed anti-Ro, anti-La, anti-Sm/RNP and anti-DNA activities but not anti-Dsg1, or -Dsg3 activity. This experiment confirmed the hypothesis that sera from pemphigus erythematosus patients possess two distinct families of autoantibodies, one corresponding to pemphigus and directed against desmosome proteins and the other directed against nucleocytoplasmic lupus antigens, such as ribonucleoproteins or deoxyribonucleoproteins (Figure 4). 

### 3.5. Evolving Immune Response in Individuals with Pemphigus Erythematosus

**Figure 5:**
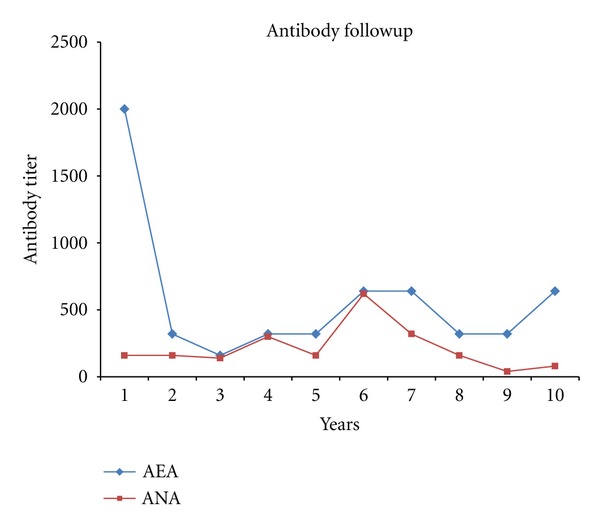
Antibody followup in three patients with pemphigus erythematosus (average of antibody titer).

To define the molecular evolution of the immune response, we tracked the immune specificity of serum from three patients over ten years. The results of this analysis yielded the conclusion that the desmoglein complex (Dsg 1) is the initial autoantibody target because antiepithelial antibodies appeared several months or years before the antinuclear response. Furthermore, the immunodominant epitope targeted the desmosome complex, as demonstrated by the antiepithelial antibody titers being higher (Figure 5). Based on this analysis, we can conclude that the desmosome complex is the initial antigenic target, and epitope spreading occurs subsequently, leading to the production of autoantibodies against ribonucleoproteins.

## 4. Discussion

The current investigation aimed to determine whether pemphigus erythematosus is a multiple-autoimmune process. The current results confirmed the coexistence of antiepithelial and antinuclear specificities in individual serum samples, as previously described [12]. Additionally, our immunochemical studies ruled out autoantibody cross-reactivity. Therefore, the present findings are relevant from the pathogenic point of view because they suggest the presence of multiple autoimmune processes in a single patient. 

Immunopathology of pemphigus erythematosus was early described by Chorzelski et al. [12] who reported the presence of immunoglobulin and complement at the dermo-epidermal junction resembling the lupus band test; their patients also exhibited antinuclear antibodies and they proposed the coexistence of pemphigus and lupus erythematosus; although this pathology was forgotten for many years, recently appeared an interesting paper by Oktarina et al. [13] who demonstrated that in pemphigus erythematosus after UV irradiation, the anti-Dsg1 antibodies were deposited along dermo-epidermal junction mimicking the lupus band in ANA negative patients, however the difference with the patients reported in present work is that they were ANA/AEA positive and they were not UV irradiated.

It is currently accepted that individuals with pemphigus vulgaris develop autoantibodies against Dsg 3, whereas in individuals with pemphigus foliaceus, the autoimmune response is targeted against Dsg 1 [14]. Because PE is considered a benign variant of pemphigus foliaceus, it is expected that these patients would develop autoantibodies against Dsg 1. However, the results of this study demonstrate that these patients simultaneously develop antibodies against desmoglein 1 and 3. This finding could be unexpected; however, the presence of antibodies against Dsg1 and 3 has been previously reported in individuals with endemic pemphigus foliaceus [15]. In the present study, we found that affinity-purified antiepithelial antibodies from eight of ten patients recognised both desmoglein 1 and 3; therefore the presence of anti-Dsg3 in our patients might result of an epitope spreading phenomenon, and its possible blistering role in PE need to be further elucidated. Based on the present results, we inferred that the autoantibodies in the patient sera were produced by independent B cell clones because the autoantibodies lacked cross-reactivity.

The concept of autoimmune overlap emerged from findings of Sharp et al., who identified a group of patients with a mixture of symptoms of lupus, scleroderma, dermatomyositis, and rheumatoid arthritis. This clinical entity was named mixed connective tissue disease (MCTD), and its biomarker was identified as the anti-RNP autoantibody [16]. However, the coexistence of different autoimmune diseases in one patient can be historically ascribed to Senear and Usher, who communicated the possible association between pemphigus and lupus [4]. Subsequently, different groups of dermatologists have described additional associations between blistering autoimmune diseases and other pathologies, such as celiac disease, thyroiditis, vitiligo, and pernicious anaemia [17–19]. The concept of shared autoimmunity is presently accepted, and, therefore, early investigations of the mechanisms involved focused on the molecular level. These studies revealed a stereochemical interaction between the hypervariable regions of MHC class II and certain epitopes [20, 21]. These studies were based on the theory proposed by Silver and Goyert in 1985 [22]. Then, the idea of a “*shared epitope*” was proposed by Gregersen et al. to explain the genetic susceptibility to rheumatoid arthritis. Interestingly, in their proposal, they noted the possible risk of the development of pemphigus vulgaris and myasthenia gravis in subjects who share epitopes in the third hypervariable region of the D locus [23]. Nevertheless, shared autoimmunity is beyond the regulatory role of the hypervariable regions of the MHC proteins, and, instead, new clues have emerged for certain alleles that define the clinical expression of autoimmunity. For example, in blistering autoimmune diseases, an allele may drive two simultaneous autoimmune responses, one directed against ribonucleoproteins and another directed against desmosome or hemidesmosome proteins. This is the case for the DQ*β*1*0301 allele in pemphigus and the DR*β*1*0402 allele in some pemphigoid varieties [24]. Theoretically, these alleles may show different stereochemical abilities to bind to two T-dependent epitopes, such as desmogleins or BP 180, and under other conditions, they might bind ribonucleoproteins. This peculiar allelic behaviour would result in a dual autoimmune response, with one organ-specific response for pemphigus and another response that manifests as a different phenotype, such as cutaneous lupus erythematosus [25–28]. This type of dual response could be involved in pemphigus erythematosus.

Based on the current findings, we can assume that the primary target of PE is the desmosomal complex because the antiepithelial antibody response appeared first, followed by the antinuclear response. Therefore, we can also infer that the desmosome is the immunodominant epitope because, in our patients, the AEA titer was always higher than the ANA titer. This immunodominance can be explained by the extracellular availability of the desmosomal complex. Finally, the molecular study of this interesting disease represents an opportunity to explore the molecular mechanisms involved in intermolecular epitope spreading as a possible cause of multiple autoimmune triggering.
